# Supplementary material for: Causal Relationship between Aging and Anorexia Nervosa: A White-Matter-Microstructure-Mediated Mendelian Randomization Analysis
Source: Biomedicines. 2024 Aug 16;12(8):1874. doi: 10.3390/biomedicines12081874 (PMC11351342; doi:10.3390/biomedicines12081874)
Supplement: Supplementary file 1 [file biomedicines-12-01874-s001.zip › Supplementary Figure/Figure S9/Leaveoneout plot of MR analysis for ubm-b-1916 to AN.pdf]

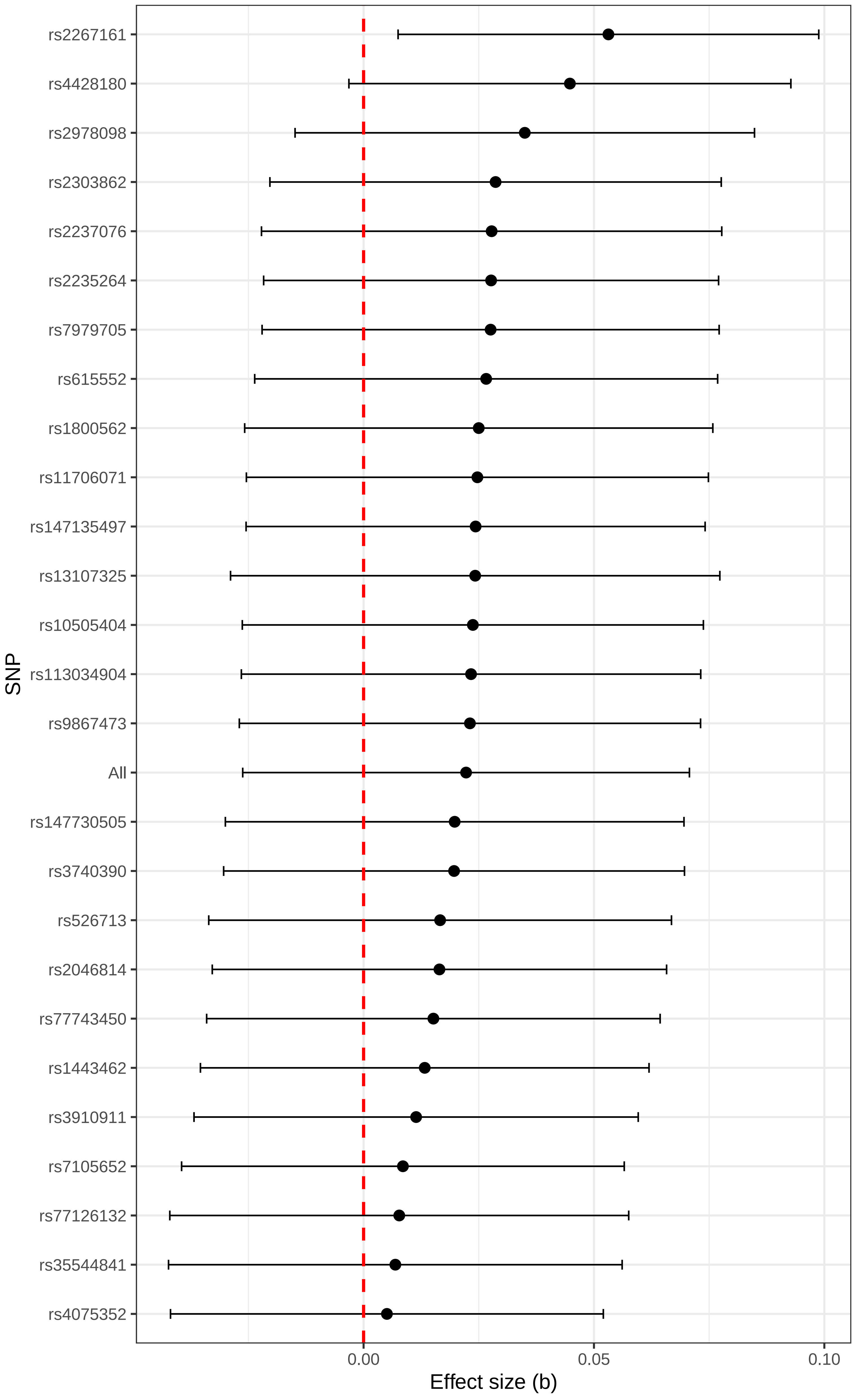
rs2267161
rs4428180
rs2978098
rs2303862
rs2237076
rs2235264
rs7979705
rs615552
rs1800562
rs11706071
rs147135497
rs13107325
rs10505404
rs113034904
rs9867473
All
rs147730505
rs3740390
rs526713
rs2046814
rs77743450
rs1443462
rs3910911
rs7105652
rs77126132
rs35544841
rs4075352
SNP
0.00
0.05
0.10
Effect size (b)
